# Layered Inter-Cluster Cooperation Scheme for Backhaul-Constrained C-RAN Uplink Systems in the Presence of Inter-Cluster Interference

**DOI:** 10.3390/e22050554

**Published:** 2020-05-15

**Authors:** Junbeom Kim, Seok-Hwan Park

**Affiliations:** Division of Electronic Engineering, Jeonbuk National University, Jeonju 54896, Korea; junbeom@jbnu.ac.kr

**Keywords:** C-RAN, inter-cluster cooperation, constrained fronthaul/backhaul, layered compression

## Abstract

Despite the potential benefits of reducing system costs and improving spectral efficiency, it is challenging to implement cloud radio access network (C-RAN) systems due to the performance degradation caused by finite-capacity fronthaul links and inter-cluster interference signals. This work studies inter-cluster cooperative reception for the uplink of a two-cluster C-RAN system, where two nearby clusters interfere with each other on the uplink access channel. The radio units (RUs) of two clusters forward quantized and compressed version of the uplink received signals to the serving baseband processing units (BBUs) via finite-capacity fronthaul links. The BBUs of the clusters exchange the received fronthaul signals via finite-capacity backhaul links with the purpose of mitigating inter-cluster interference signals. Optimization of conventional cooperation scheme, in which each RU produces a single quantized signal, requires an exhaustive discrete search of exponentially increasing search size with respect to the number of RUs. To resolve this issue, we propose an improved inter-BBU, or inter-cluster, cooperation strategy based on layered compression, where each RU produces two descriptions, of which only one description is forwarded to the neighboring BBU on the backhaul links. We discuss the optimization of the proposed inter-cluster cooperation scheme, and validate the performance gains of the proposed scheme via numerical results.

## 1. Introduction

Cloud radio access network (C-RAN) systems have a potential of reducing the capital and operating expenditures and of improving spectral and energy efficiency. These benefits can be realized by centralized baseband signal processing at baseband processing unit (BBU) pools [1,2,3]. However, it is challenging to reliably transfer baseband samples on fronthaul links that connect distributed radio units (RUs) to nearby BBUs particularly for broadband communication systems. To address this issue, the authors of [4,5] proposed efficient compression techniques which can effectively reduce the fronthaul overhead by exploiting signal correlation among distributed RUs. Signal processing design of fronthaul-constrained C-RAN systems has also been studied in more complicated C-RAN systems that are equipped with multi-hop fronthaul networks [6] or with spectrum pooling capability among network operators [7].

Another challenge to implement C-RAN is that it is not trivial to mitigate the impact of interference signals among nearby clusters, where each cluster consists of a set of RUs and users that are served by a single BBU. Dynamic clustering approaches based on instantaneous channel state information (CSI) were proposed and analyzed in [8,9]. For given clusters, the authors of [10] addressed inter-cluster coordinated design of downlink precoding and fronthaul compression strategies, and investigated the advantages of inter-cluster coordinated design compared to inter-cluster time-division multiple access (TDMA) or intra-cluster design which neglects the impact of inter-cluster interference signals.

In this work, we propose an inter-cluster, or inter-BBU, cooperative reception strategy that aims at mitigating the impact of inter-cluster interference signals in the uplink of C-RAN systems. We consider a practical inter-cluster cooperation model, in which the BBUs of two nearby clusters exchange the information of in-cluster uplink baseband signals on finite-capacity backhaul links. In the conventional inter-BBU cooperation scheme proposed in [7], each RU produces a single quantized signal, or single description, and one needs to decide the set of RUs whose quantized signals are transferred not only to the serving BBU but also to the neighboring BBU. The optimization of this scheme asks for a discrete search of exponentially increasing search size with respect to the number of RUs.

Motivated by this issue, we propose a layered compression strategy at RUs, whereby each RU produces two quantized signals that are decompressed only by the serving BBU or both by the serving and neighboring BBUs, and the compression rate allocation among the two descriptions is included to the design space. With this approach, we can efficiently utilize the fronthaul and backhaul links without resorting to a discrete search. Similar approaches were studied in [11,12] that adopt a layered compression strategy for robust exploitation of packet-based fronthaul networks [11] or for flexible inter-user cooperation [12]. It was reported by [11] that multiple description coding can outperform traditional packet diversity techniques in terms of efficiently utilizing multiple routes, which are subject to independent congestion and packet losses, in packet-based multi-hop fronthaul networks. [12] investigated the advantages of broadcast coding and layered compression under the scenario of inter-user cooperation, in which a user informs multiple users through a broadcast channel with different channel gains across receiving users. We note that in the studies of [11,12], multiple description coding was used to enable compression fidelity to be adapted to different packet loss events or different channel gains. Unlike those, in this work, we adopt multiple description coding with the aim of making the quality of the quantized signals decompressed at serving and neighboring BBUs different from each other, since the RU-to-BBU fronthaul and inter-BBU backhaul links can have different capacity.

The paper is organized as follows. In Section 2, we describe the uplink of a two-cluster C-RAN system. In Section 3, we review baseline uplink reception strategies with no or conventional inter-BBU cooperation strategies. We propose an improved cooperation scheme based on layered compression in Section 4, where we also discuss the signal processing optimization of the proposed scheme. In Section 5, we provide numerical results that check the convergence property of the proposed algorithm and the performance gains of the proposed scheme compared to the baseline schemes discussed in Section 3. We close the paper in Section 6.

Throughout the paper, we use the following notations. The circularly symmetric complex Gaussian distribution with zero mean and variance $\sigma^{2}$ is denoted by $\mathrm{CN}(0,\sigma^{2})$. I(X;Y) denotes the mutual information between two random variables *X* and *Y*. The transpose and Hermitian transpose of a vector or matrix are denoted by ${\left(\cdot \right)}^{T}$ and ${\left(\cdot \right)}^{H}$, respectively, and $C^{M\times N}$ denotes the set of all *M*-by*N* complex matrices. We denote the Euclidean 2-norm of a vector by $\left|\right|\cdot {\left|\right|}^{2}$.

## 2. System Model

**Figure 1:**
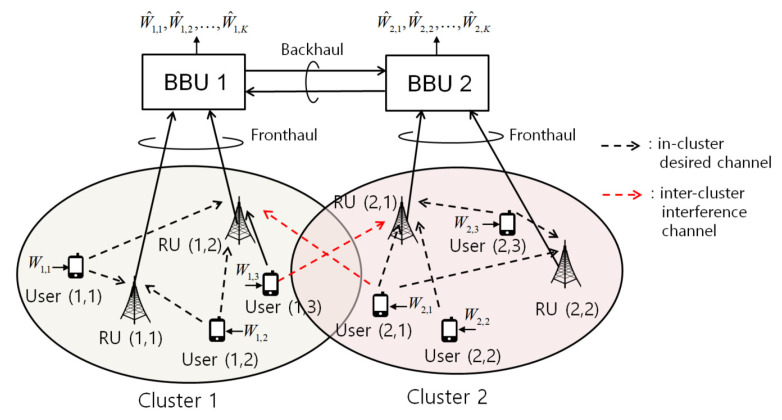
Illustration of the uplink of a two-cluster C-RAN system, in which two neighboring clusters interfere with each other on the uplink channel and the BBUs cooperate via backhaul links.

We consider the uplink of a two-cluster C-RAN system illustrated in Figure 1. The system consists of two nearby clusters, where each cluster has *K* single-antenna users, *M* single-antenna RUs, and a single BBU. There are no overlapped users, RUs, and BBUs between the two clusters. We refer to the *k*th user, the *r*th RU, and the BBU in cluster *i* as user (i,k), RU (i,r), and BBU *i*, respectively. The users (i,k), k∈K≜{1,2,…,K}, in cluster *i* transmit digital messages to their serving BBU *i* through the RUs (i,r), r∈M≜{1,2,…,M}. Each RU (i,r) is connected to BBU *i* through a *fronthaul link* of capacity $C_{F}$ bit/symbol. To efficiently manage inter-cluster interference signals, each BBU *i* can send some information to BBU $\bar{i}≜3-i$ through a *backhaul link* of finite capacity $C_{B}$ bit/symbol. We assume that the association between users and clusters is given a priori, and the design of association is left as a future work.

### 2.1. Users-to-RUs Uplink Channel Model

We denote the received signal of RU (i,r) by $y_{i,r}$ which can be written under flat-fading channel model as

(1) $$\begin{array}{c} y_{i,r}=\sum_{k\in K}h_{i,r,k}x_{i,k}+\sum_{k\in K}g_{i,r,k}x_{\bar{i},k}+z_{i,r}. \end{array}$$

Here, $x_{i,k}$ denotes the transmit signal of user (i,k) and satisfies a transmit power constraint $E[|x_{i,k}{|}^{2}]\leq P$ with *P* denoting the power budget of each user. $h_{i,r,k}$ represents the channel coefficient from user (i,k) to RU (i,r), $g_{i,r,k}$ is the channel coefficient from user $\left(\bar{i},k\right)$ to RU (i,r), and $z_{i,r}$ indicates the noise signal at RU (i,r) with $z_{i,r}∼\mathrm{CN}\left(0,\sigma_{z}^{2}\right)$. On the right-hand side (RHS) of Equation (1), the first term indicates the desired signal transmitted by the in-cluster users (i,k), i∈K, and the second term represents the interference signals from the neighboring cluster’s users $\left(\bar{i},k\right)$, k∈K.

### 2.2. Channel Encoding at Users

We denote the message of user (i,k) by $W_{i,k}$ whose rate is $R_{i,k}$ bit/symbol. BBU *i* tries to decode the messages $W_{i,1},W_{i,2},\ldots ,W_{i,K}$ of in-cluster users. User (i,k) performs channel encoding with Gaussian channel codebook so that the transmit signal $x_{i,k}$, which encodes $W_{i,k}$, follows the distribution $x_{i,k}∼\mathrm{CN}\left(0,P\right)$, i.e., $E[|x_{i,k}{|}^{2}]=P$. We define the uplink signal-to-noise ratio (SNR) as $P/\sigma_{z}^{2}$. We also note that dynamic power control at users, instead of fixed full power transmission, may improve the performance with additional overhead for CSI acquisition at users.

## 3. Conventional Uplink Reception Strategies

In this section, we describe the uplink reception without inter-cluster cooperation [5,6] or with a conventional inter-cluster cooperation strategy [7]. Each RU (i,r) needs to send the information of the uplink received signal $y_{i,r}$ to the serving BBU *i*. Due to the fronthaul capacity limitation, RU (i,r) quantizes the signal and sends the resulting bit stream on the fronthaul link. Following the results from standard rate distortion theory [13], the quantized signal represented by the bit stream can be modeled as

(2) $$\begin{array}{c} {\hat{y}}_{i,r}=y_{i,r}+q_{i,r}, \end{array}$$

where the quantization distortion $q_{i,r}$ is independent of $y_{i,r}$ and distributed as $q_{i,r}∼\mathrm{CN}\left(0,\omega_{i,r}\right)$ under the Gaussian test channel [5,7]. Here, $\omega_{i,r}$ stands for the quantization noise power with $\omega_{i,r}>0$.

In this work, we focus on a point-to-point compression strategy [7,14,15], in which BBU *i* decompresses the quantized signals ${\hat{y}}_{i,1},{\hat{y}}_{i,2},\ldots ,{\hat{y}}_{i,M}$ separately (the analysis and design with more sophisticated BBU operations, such as successive decompression [4] or joint decompression and decoding [5], is left as a future work). Under this assumption, the distorted signal ${\hat{y}}_{i,r}$ after quantization can be recovered by BBU *i* based on the bit stream received on the fronthaul link, if the following condition is satisfied [13].

(3) $$\begin{array}{c} I\left(y_{i,r};{\hat{y}}_{i,r}\right)=\log_{2}\left(1+\frac{\sigma_{y,i,r}^{2}}{\omega_{i,r}}\right)\leq C_{F}, \end{array}$$

where the power $\sigma_{y,i,r}^{2}$ of $y_{i,r}$ is given as

(4) $$\begin{array}{c} \sigma_{y,i,r}^{2}=\sum_{k\in K}P|h_{i,r,k}{|}^{2}+\sum_{k\in K}P{\left|g_{i,r,k}\right|}^{2}+\sigma_{z}^{2}. \end{array}$$

### 3.1. No Inter-BBU Cooperation

In this subsection, we review the conventional scheme without inter-BBU cooperation, in which each BBU *i* decodes the messages $W_{i,1},W_{i,2},\ldots ,W_{i,K}$ sent by in-cluster users by exploiting only the signals ${\hat{y}}_{i,1},{\hat{y}}_{i,2},\ldots ,{\hat{y}}_{i,M}$ received on the fronthaul links. With this approach, the backhaul links connecting the BBUs are not utilized at all.

If we assume that BBU *i* decodes the messages $W_{i,1},W_{i,2},\ldots ,W_{i,K}$ while treating the interference signals $x_{\bar{i},1},x_{\bar{i},2},\ldots ,x_{\bar{i},K}$ from the other cluster $\bar{i}$ as noise, the achievable sum-rate $R_{\mathrm{sum},i}≜\sum_{k\in K}R_{i,k}$ of cluster *i* is given as

(5) $$\begin{array}{c} R_{\mathrm{sum},i}=I\left(x_{i};{\hat{y}}_{i}\right)=\log_{2}\det\left(I+P{\left(PG_{i}G_{i}^{H}+\sigma_{z}^{2}I+\Omega_{i}\right)}^{-1}H_{i}H_{i}^{H}\right), \end{array}$$

where we define the notations $x_{i}≜{\left[x_{i,1}x_{i,2}\cdots x_{i,K}\right]}^{T}$, ${\hat{y}}_{i}≜{\left[{\hat{y}}_{i,1}{\hat{y}}_{i,2}\cdots {\hat{y}}_{i,M}\right]}^{T}$, $\Omega_{i}≜\mathrm{diag}\left({\left\{\omega_{i,r}\right\}}_{r\in M}\right)$, and the (r,k)th elements of $H_{i}\in C^{M\times K}$ and $G_{i}\in C^{M\times K}$ are given as $h_{i,r,k}$ and $g_{i,r,k}$, respectively.

Note that, in this approach that does not employ inter-BBU cooperation, the optimal quantization power $\omega_{i,r}$ is simply the minimum value that satisfies the condition in Equation (3), since there is no overhead of the backhaul links. Such minimum value of $\omega_{i,r}$ is given as

(6) $$\begin{array}{c} \omega_{i,r}=\frac{\sigma_{y,i,r}^{2}}{2^{C_{F}}-1}. \end{array}$$

**Remark** **1.** *Suppose that BBU i decodes and cancels the interference signals $x_{\bar{i},k}$, $k\in {\tilde{K}}_{\bar{i}}$, from the neighboring cluster $\bar{i}$ prior to decoding the desired in-cluster signals. Then, the sum-rate $R_{\mathrm{sum},i}$ of cluster i is bounded as*

(7) $$\begin{array}{c} R_{\mathrm{sum},i}\leq \log_{2}\det\left(I+P{\left(\sum_{k\in K\backslash {\tilde{K}}_{\bar{i}}}Pg_{i,k}g_{i,k}^{H}+\sigma_{z}^{2}I+\Omega_{i}\right)}^{-1}H_{i}H_{i}^{H}\right), \end{array}$$

*where $g_{i,k}\in C^{M\times 1}$ denotes the kth column vector of $G_{i}$. Note that, as long as ${\tilde{K}}_{\bar{i}}$ is not empty, the RHS of Equation (7) is strictly larger than that of Equation (5), since the interference covariance terms have been reduced. However, unlike Equation (5), the condition in Equation (7) may not be satisfied with equality, since the rates $R_{i,k}$ with $k\in {\tilde{K}}_{i}$ are subject to additional constraints for successful interference decoding at the neighboring BBU $\bar{i}$. This suggests that the sets ${\tilde{K}}_{1}$ and ${\tilde{K}}_{2}$ of decoded interference signals need to be carefully chosen depending on the SNR $P/\sigma_{z}^{2}$ as well as the instantaneous CSI. An exhaustive search for finding the optimal sets ${\tilde{K}}_{1}$ and ${\tilde{K}}_{2}$ requires a search size exponentially increasing with K. This calls for the development of an efficient selection algorithm that achieves a good trade-off between the performance and complexity.*

### 3.2. Conventional Inter-BBU Cooperation

This subsection discusses the conventional inter-BBU cooperation scheme [7], where each BBU *i* sends a selected subset of the signals ${\hat{y}}_{i,1},{\hat{y}}_{i,2},\ldots ,{\hat{y}}_{i,M}$ to the other BBU $\bar{i}$. We note that it may not be optimal to send all the signals on the backhaul link, which has a limited capacity $C_{B}$, particularly when the capacity $C_{B}$ of backhaul links is much smaller than $C_{F}$. We define ${\tilde{M}}_{i}\subseteq M$ as the set of RUs’ indices whose quantized signals are transferred to BBU $\bar{i}$ on the backhaul link. Without claim of optimality, following the policy proposed in [7], we fill each set ${\tilde{M}}_{i}$ with the indices of $\tilde{M}$ RUs that have the largest channel gains from the users in the neighboring cluster $\bar{i}$, i.e.,

(8) $$\begin{array}{c} {\tilde{M}}_{i}=\left\{r_{i,1},r_{i,2},\ldots ,r_{i,\tilde{M}}\right\}, \end{array}$$

where we have $\left|\right|g_{i,r_{1}}{\left|\right|}^{2}\geq \left|\right|g_{i,r_{2}}{\left|\right|}^{2}\geq \ldots \geq \left|\right|g_{i,r_{M}}{\left|\right|}^{2}$ with $g_{i,r}≜{\left[g_{i,r,1}g_{i,r,2}\cdots g_{i,r,K}\right]}^{T}$. The number $\tilde{M}\in \left\{0,1,\ldots ,M\right\}$ determines the level of inter-cluster cooperation, and setting $\tilde{M}=0$ leads to the no cooperation scheme discussed in Section 3.1. In principle, increasing the level $\tilde{M}$ enables stronger cooperation among the BBUs. However, when the backhaul links have small capacity, large $\tilde{M}$ degrades the resolution of the quantized signals to satisfy the backhaul capacity constraints, and the fronthaul links are not fully utilized. Therefore, the optimal level $\tilde{M}$ should be carefully chosen depending on the instantaneous channel states as well as the backhaul and fronthaul capacities.

We denote the compression rate allocated to express the signal ${\hat{y}}_{i,r}$ by $R_{C,i,r}\in \left[0,C_{F}\right]$, and the rates ${\left\{R_{C,i,r}\right\}}_{r\in {\tilde{M}}_{i}}$ should satisfy the condition

(9) $$\begin{array}{c} \sum_{r\in {\tilde{M}}_{i}}R_{C,i,r}\leq C_{B}. \end{array}$$

In addition, with this approach, the upper-threshold $C_{F}$ in the RHS of the fronthaul capacity constraint in Equation (3) is replaced with $R_{C,i,r}$ for RUs (i,r) with $r\in {\tilde{M}}_{i}$. This is because, if $R_{C,i,r}<C_{F}$, we can use only partial, instead of full, capacity of those RUs’ fronthaul links, since the quantized signals should be transferred on the fronthaul link to BBU *i* as well as on the backhaul link to the other BBU $\bar{i}$.

We assume that BBU *i* decodes the messages $W_{i,1},W_{i,2},\ldots ,W_{i,K}$ by leveraging the signals ${\hat{y}}_{i}$ received on the fronthaul links from in-cluster RUs as well as the signals ${\hat{y}}_{\bar{i},r_{\bar{i},1}},{\hat{y}}_{\bar{i},r_{\bar{i},2}},\ldots ,{\hat{y}}_{\bar{i},r_{\bar{i},\tilde{M}}}$ received on the backhaul link from the other BBU $\bar{i}$. Then, the achievable sum-rate $R_{\mathrm{sum},i}$ of cluster *i* is given as

(10) $$\begin{array}{cc} R_{\mathrm{sum},i} & =I\left(x_{i};\;\;{\hat{y}}_{i},{\left\{{\hat{y}}_{\bar{i},r_{\bar{i},r}}\right\}}_{r\in {\tilde{M}}_{\hat{i}}}\right)\\ \; & =\log_{2}\det\left(I+P{\left(P{\tilde{G}}_{i}{\tilde{G}}_{i}^{H}+\sigma_{z}^{2}I+{\tilde{\Omega }}_{i}\right)}^{-1}{\tilde{H}}_{i}{\tilde{H}}_{i}^{H}\right), \end{array}$$

where we define the matrices ${\tilde{H}}_{i}\in C^{(M+\tilde{M})\times K}$, ${\tilde{G}}_{i}\in C^{(M+\tilde{M})\times K}$ and ${\tilde{\Omega }}_{i}\in C^{\left(M+\tilde{M}\right)\times \left(M+\tilde{M}\right)}$ as

(11) $$\begin{array}{c} {\tilde{H}}_{i}=\left[\begin{array}{c} H_{i}\\ \left[\begin{array}{c} e_{r_{\bar{i},1}}^{H}\\ \vdots \\ e_{r_{\bar{i},\tilde{M}}}^{H} \end{array}\right]G_{\bar{i}} \end{array}\right],\;\;{\tilde{G}}_{i}=\left[\begin{array}{c} G_{i}\\ \left[\begin{array}{c} e_{r_{\bar{i},1}}^{H}\\ \vdots \\ e_{r_{\bar{i},\tilde{M}}}^{H} \end{array}\right]H_{\bar{i}} \end{array}\right],\;\;{\tilde{\Omega }}_{i}=\mathrm{diag}\left({\left\{\omega_{i,r}\right\}}_{r\in M},{\left\{\omega_{\bar{i},r_{\bar{i},l}}\right\}}_{l=1}^{\tilde{M}}\right), \end{array}$$

with $e_{r}\in C^{M\times 1}$ defined as a column vector filled with zeros except for the *r*th element, which equals 1.

We omit the discussion on the optimization of the quantization noise powers ${\left\{\omega_{i,r}\right\}}_{i\in \{1,2\},r\in M}$ for fixed sets ${\tilde{M}}_{1}$ and ${\tilde{M}}_{2}$, since it can be handled in a similar way to the optimization of the proposed scheme that is discussed in the next section.

## 4. Uplink Reception With Proposed Cooperation

In this section, we propose an improved inter-BBU cooperation scheme based on layered compression, or successive refinement quantization, strategy [16,17] (see also [13]). In this approach, each RU (i,r) is equipped with two compression encoders, where the *j*th encoder generates a description $U_{i,r,j}$ of compression rate $R_{C,i,r,j}$ bit/symbol by quantizing and compressing the received signal $y_{i,r}$ of RU (i,r). The description $U_{i,r,1}$ is referred to as *basement layer* from which a quantized signal ${\hat{y}}_{i,r,1}$ can be recovered. The other description $U_{i,r,2}$ is referred to as *enhancement layer*, since a better reconstruction ${\hat{y}}_{i,r,2}$ can be obtained by using both descriptions $U_{i,r,1}$ and $U_{i,r,2}$, i.e., $y_{i,r}↔{\hat{y}}_{i,r,2}↔{\hat{y}}_{i,r,1}$.

Following [12], we assume the Gaussian test channel for both ${\hat{y}}_{i,r,1}$ and ${\hat{y}}_{i,r,2}$, i.e.,

(12) $$\begin{array}{c} {\hat{y}}_{i,r,j}=y_{i,r}+q_{i,r,j}, \end{array}$$

with the quantization noise $q_{i,r,j}∼\mathrm{CN}\left(0,\omega_{i,r,j}\right)$ being independent of $y_{i,r}$. In order for the descriptions ${\hat{y}}_{i,r,1}$ and ${\hat{y}}_{i,r,2}$ to be successfully recovered, the following conditions should be satisfied.

(13) $$\begin{array}{c} I\left(y_{i,r};{\hat{y}}_{i,r,j}\right)=\log_{2}\left(1+\frac{\sigma_{y,i,r}^{2}}{\omega_{i,r,j}}\right)\leq \sum_{m=1}^{j}R_{C,i,r,m}, \end{array}$$

for j∈{1,2}.

To enable a flexible inter-BBU cooperation, we assume that the basement layer $U_{i,r,1}$ of each RU (i,r) is transferred to both the serving BBU *i* and the neighboring BBU $\bar{i}$, while the enhancement layer $U_{i,r,2}$ is delivered only to the serving BBU *i*. Therefore, BBU *i* can recover the quantized signal ${\hat{y}}_{i,r,2}$, which better represents the received signal $y_{i,r}$ of RU (i,r) than the other quantized signal ${\hat{y}}_{i,r,1}$ reconstructed at BBU $\bar{i}$ does. Under the assumption that we use the El Gamal-Cover coding scheme [18] for the compression encoders and decoders, the described process can be made possible if the compression rates $R_{C,i,r,j}$ satisfy the following constraints.

(14) $$\begin{array}{c} R_{C,i,r,1}+R_{C,i,r,2}\leq C_{F},\;\;\mathrm{for}\;\;i\in \left\{1,2\right\},r\in M, \end{array}$$

(15) $$\begin{array}{c} \sum_{r\in M}R_{C,i,r,1}\leq C_{B},\;\;\mathrm{for}\;\;i\in \left\{1,2\right\}. \end{array}$$

We refer to ([13], Section 13.5) for the detailed proof.

We assume that each BBU *i* decodes the messages $W_{i,1},W_{i,2},\ldots ,W_{i,K}$ from the high-resolution quantized signals ${\hat{y}}_{i,2}≜{\left[{\hat{y}}_{i,1,2}{\hat{y}}_{i,2,2}\cdots {\hat{y}}_{i,M,2}\right]}^{T}$ associated with in-cluster RUs and the low-resolution quantized signals ${\hat{y}}_{\bar{i},1}≜{\left[{\hat{y}}_{\bar{i},1,1}{\hat{y}}_{\bar{i},2,1}\cdots {\hat{y}}_{\bar{i},M,1}\right]}^{T}$ corresponding to the other-cluster RUs. The achievable sum-rate $R_{\mathrm{sum},i}$ of cluster *i* is given as

(16) $$\begin{array}{cc} R_{\mathrm{sum},i} & =I\left(x_{i};\;\;{\hat{y}}_{i,2},{\hat{y}}_{\bar{i},1}\right)\\ \; & =\log_{2}\det\left(I+P{\left(P{\overset{ˇ}{G}}_{i}{\overset{ˇ}{G}}_{i}^{H}+\sigma_{z}^{2}I+{\overset{ˇ}{\Omega }}_{i}\right)}^{-1}{\overset{ˇ}{H}}_{i}{\overset{ˇ}{H}}_{i}^{H}\right), \end{array}$$

where we define the matrices ${\overset{ˇ}{H}}_{i}\in C^{2M\times K}$, ${\overset{ˇ}{G}}_{i}\in C^{2M\times K}$ and ${\overset{ˇ}{\Omega }}_{i}\in C^{2M\times 2M}$ as

(17) $$\begin{array}{c} {\overset{ˇ}{H}}_{i}=\left[\begin{array}{c} H_{i}\\ G_{\bar{i}} \end{array}\right],\;{\overset{ˇ}{G}}_{i}=\left[\begin{array}{c} G_{i}\\ H_{\bar{i}} \end{array}\right],\;{\overset{ˇ}{\Omega }}_{i}=\mathrm{diag}\left({\left\{\omega_{i,r,2}\right\}}_{r\in M},{\left\{\omega_{\bar{i},r,1}\right\}}_{r\in M}\right). \end{array}$$

We now discuss the optimization of the proposed layered inter-cluster cooperation strategy. We aim at maximizing the sum-rate $R_{\mathrm{sum}}≜\sum_{i\in \{1,2\}}R_{\mathrm{sum},i}$ of all the users in the clusters while satisfying the fronthaul and backhaul capacity constraints. The problem can be mathematically formulated as

(18a) $$\begin{array}{cc} \underset{\omega ,R_{C},R_{\mathrm{sum}}}{\mathrm{maximize}}\;\;\; & \sum_{i\in \{1,2\}}R_{\mathrm{sum},i} \end{array}$$

(18b) $$\begin{array}{cc} s.t.\;\;\; & R_{\mathrm{sum},i}\leq \log_{2}\det\left(I+P{\left(P{\overset{ˇ}{G}}_{i}{\overset{ˇ}{G}}_{i}^{H}+\sigma_{z}^{2}I+{\overset{ˇ}{\Omega }}_{i}\right)}^{-1}{\overset{ˇ}{H}}_{i}{\overset{ˇ}{H}}_{i}^{H}\right),\;\;\forall i\in \left\{1,2\right\}, \end{array}$$

(18c) $$\begin{array}{c} \log_{2}\left(1+\frac{\sigma_{y,i,r}^{2}}{\omega_{i,r,j}}\right)\leq \sum_{m=1}^{j}R_{C,i,r,m},\;\;\forall i\in \left\{1,2\right\},\;r\in M,\;j\in \left\{1,2\right\}, \end{array}$$

(18d) $$\begin{array}{c} R_{C,i,r,1}+R_{C,i,r,2}\leq C_{F},\;\;\forall i\in \left\{1,2\right\},r\in M, \end{array}$$

(18e) $$\begin{array}{c} \sum_{r\in M}R_{C,i,r,1}\leq C_{B},\;\;\forall i\in \left\{1,2\right\}, \end{array}$$

(18f) $$\begin{array}{c} R_{C,i,r,j}\geq 0,\;\;\omega_{i,r,j}>0,\;\;\forall i\in \left\{1,2\right\},\;r\in M,\;j\in \left\{1,2\right\}, \end{array}$$

where we define the notations $\omega ≜{\left\{\omega_{i,r,j}\right\}}_{i\in \{1,2\},r\in M,j\in \{1,2\}}$, $R_{C}≜{\left\{R_{C,i,r,j}\right\}}_{i\in \{1,2\},r\in M,j\in \{1,2\}}$, and $R_{\mathrm{sum}}≜{\left\{R_{\mathrm{sum},i}\right\}}_{i\in \{1,2\}}$.

It is challenging to find an optimal solution of the problem in Equation (18), since it is a non-convex problem due to the constraints in Equations (18b) and (18c). However, we can obtain a difference-of-convex (DC) problem, whose objective and constraint functions can be expressed as differences of convex functions, by replacing Equations (18b) and (18c) with the following equivalent constraints

(19) $$\begin{array}{cc} R_{\mathrm{sum},i} & \leq \log_{2}\det\left(P{\overset{ˇ}{G}}_{i}{\overset{ˇ}{G}}_{i}^{H}+\sigma_{z}^{2}I+{\overset{ˇ}{\Omega }}_{i}+P{\overset{ˇ}{H}}_{i}{\overset{ˇ}{H}}_{i}^{H}\right)\\ \; & -\log_{2}\det\left(P{\overset{ˇ}{G}}_{i}{\overset{ˇ}{G}}_{i}^{H}+\sigma_{z}^{2}I+{\overset{ˇ}{\Omega }}_{i}\right),\;\;\forall i\in \left\{1,2\right\}, \end{array}$$

and

(20) $$\begin{array}{cc} \sum_{m=1}^{j}R_{C,i,r,m} & \geq \log_{2}\left(\omega_{i,r,j}+\sigma_{y,i,r}^{2}\right)\\ \; & -\log_{2}\omega_{i,r,j},\;\;\forall i\in \left\{1,2\right\},\;r\in M,\;j\in \left\{1,2\right\}. \end{array}$$

A desirable property of the DC problems is that a locally optimal solution can be efficiently found via a Majorization Minimization (MM) based iterative algorithm (see, e.g., [19,20]).

The MM approach can be applied to tackle the problem at hand as follows: Suppose that the constraints in Equations (19) and (20), which are equivalent to Equations (18b) and (18c), are satisfied with setting $\omega_{i,r,j}=\omega_{i,r,j}^{\prime }$, i∈{1,2},r∈M,j∈{1,2}. Then, we consider the following conditions obtained by replacing the second and first terms on the RHSs of Equations (19) and (20), respectively, with their first-order Taylor approximations with the reference points $\omega^{\prime }≜{\left\{\omega_{i,r,j}^{\prime }\right\}}_{i\in \{1,2\},r\in M,j\in \{1,2\}}$:

(21) $$\begin{array}{cc} R_{\mathrm{sum},i} & \leq \log_{2}\det\left(P{\overset{ˇ}{G}}_{i}{\overset{ˇ}{G}}_{i}^{H}+\sigma_{z}^{2}I+{\overset{ˇ}{\Omega }}_{i}+P{\overset{ˇ}{H}}_{i}{\overset{ˇ}{H}}_{i}^{H}\right)\\ \; & -\log_{2}\det\left(P{\overset{ˇ}{G}}_{i}{\overset{ˇ}{G}}_{i}^{H}+\sigma_{z}^{2}I+{\overset{ˇ}{\Omega }}_{i}^{\prime }\right)\\ \; & -\frac{1}{\ln2}\mathrm{tr}\left({\left(P{\overset{ˇ}{G}}_{i}{\overset{ˇ}{G}}_{i}^{H}+\sigma_{z}^{2}I+{\overset{ˇ}{\Omega }}_{i}^{\prime }\right)}^{-1}\left({\overset{ˇ}{\Omega }}_{i}-{\overset{ˇ}{\Omega }}_{i}^{\prime }\right)\right),\;\;\forall i\in \left\{1,2\right\}, \end{array}$$

(22) $$\begin{array}{cc} \sum_{m=1}^{j}R_{C,i,r,m} & \geq \log_{2}\left(\omega_{i,r,j}^{\prime }+\sigma_{y,i,r}^{2}\right)+\frac{1}{\ln2}\frac{1}{\omega_{i,r,j}^{\prime }+\sigma_{y,i,r}^{2}}\left(\omega_{i,r,j}-\omega_{i,r,j}^{\prime }\right)\\ \; & -\log_{2}\omega_{i,r,j},\;\;\forall i\in \left\{1,2\right\},\;r\in M,\;j\in \left\{1,2\right\}. \end{array}$$

Note that the feasible set for the constraints in Equations (21) and (22) is convex and not empty, since the non-convexity-inducing terms in Equations (19) and (20) have been linearized, and at least the point $\omega^{\prime }$ belongs to the feasible space of Equations (21) and (22). Moreover, the constraints in Equations (21) and (22) are stricter than those in Equations (19) and (20) due to the following inequalities:

(23) $$\begin{array}{cc} \log_{2}\det\left(P{\overset{ˇ}{G}}_{i}{\overset{ˇ}{G}}_{i}^{H}+\sigma_{z}^{2}I+{\overset{ˇ}{\Omega }}_{i}\right)\; & \leq \log_{2}\det\left(P{\overset{ˇ}{G}}_{i}{\overset{ˇ}{G}}_{i}^{H}+\sigma_{z}^{2}I+{\overset{ˇ}{\Omega }}_{i}^{\prime }\right)\\ \; & -\frac{1}{\ln2}\mathrm{tr}\left({\left(P{\overset{ˇ}{G}}_{i}{\overset{ˇ}{G}}_{i}^{H}+\sigma_{z}^{2}I+{\overset{ˇ}{\Omega }}_{i}^{\prime }\right)}^{-1}\left({\overset{ˇ}{\Omega }}_{i}-{\overset{ˇ}{\Omega }}_{i}^{\prime }\right)\right), \end{array}$$

(24) $$\begin{array}{cc} \log_{2}\left(\omega_{i,r,j}+\sigma_{y,i,r}^{2}\right) & \leq \log_{2}\left(\omega_{i,r,j}^{\prime }+\sigma_{y,i,r}^{2}\right)+\frac{1}{\ln2}\frac{1}{\omega_{i,r,j}^{\prime }+\sigma_{y,i,r}^{2}}\left(\omega_{i,r,j}-\omega_{i,r,j}^{\prime }\right). \end{array}$$

This means that, if we find a solution to the *convex* problem, which is obtained by replacing Equations (18b) and (18c) with Equations (21) and (22) in Equation (18), the resulting solution, denoted by $\omega^{\prime \prime }≜{\left\{\omega_{i,r,j}^{\prime \prime }\right\}}_{i\in \{1,2\},r\in M,j\in \{1,2\}}$, will achieve a sum-rate larger than or equal to that of $\omega^{\prime }$, while satisfying all the constraints of the original problem in Equation (18). If the sum-rate improvement is not negligible, we do the same process after updating the reference point $\omega^{\prime }\leftarrow \omega^{\prime \prime }$, and this process can be repeated until the sum-rate converges. Since the sum-rate monotonically increases with iterations and the optimal sum-rate of the problem in Equation (18) is finite, the convergence of the MM algorithm is guaranteed. We refer to [21,22] for more formal proof of convergence and stability of the MM algorithms. The MM algorithm customized to solve our DC problem is described in Algorithm 1.

**Algorithm 1** MM algorithm for optimizing ω, $R_{C}$ and $R_{\mathrm{sum}}$,**1.** Initialize $R_{C}$ as arbitrary rates that satisfy the constraints in Equations (18d)–(18f).**2.** Set $\omega_{i,r,j}^{\prime }$ to $\omega_{i,r,j}^{\prime }\leftarrow \sigma_{y,i,r}^{2}/\left(2^{\sum_{m=1}^{j}R_{C,i,r,m}}-1\right)$, i.e., the minimum value that satisfies the constraint in Equation (18c), for i∈{1,2}, r∈M, j∈{1,2}.**3.** Update ω as a solution of the convex problem:
(25a)$$\begin{array}{cc} \underset{\omega ,R_{C},R_{\mathrm{sum}}}{\mathrm{maximize}}\;\;\; & \sum_{i\in \{1,2\}}R_{\mathrm{sum},i} \end{array}$$

(25b)$$\begin{array}{cc} s.t.\;\;\; & R_{\mathrm{sum},i}\leq \log_{2}\det\left(P{\overset{ˇ}{G}}_{i}{\overset{ˇ}{G}}_{i}^{H}+\sigma_{z}^{2}I+{\overset{ˇ}{\Omega }}_{i}+P{\overset{ˇ}{H}}_{i}{\overset{ˇ}{H}}_{i}^{H}\right)\\ \; & -\log_{2}\det\left(P{\overset{ˇ}{G}}_{i}{\overset{ˇ}{G}}_{i}^{H}+\sigma_{z}^{2}I+{\overset{ˇ}{\Omega }}_{i}^{\prime }\right)\\ \; & -\frac{1}{\ln2}\mathrm{tr}\left({\left(P{\overset{ˇ}{G}}_{i}{\overset{ˇ}{G}}_{i}^{H}+\sigma_{z}^{2}I+{\overset{ˇ}{\Omega }}_{i}^{\prime }\right)}^{-1}\left({\overset{ˇ}{\Omega }}_{i}-{\overset{ˇ}{\Omega }}_{i}^{\prime }\right)\right),\;\;\forall i\in \left\{1,2\right\}, \end{array}$$

(25c)$$\begin{array}{c} \sum_{m=1}^{j}R_{C,i,r,m}\geq \log_{2}\left(\omega_{i,r,j}^{\prime }+\sigma_{y,i,r}^{2}\right)+\frac{1}{\ln2}\frac{1}{\omega_{i,r,j}^{\prime }+\sigma_{y,i,r}^{2}}\left(\omega_{i,r,j}-\omega_{i,r,j}^{\prime }\right)\\ \;\;\;-\log_{2}\;\;\omega_{i,r,j},\;\;\forall i\in \left\{1,2\right\},\;r\in M,\;j\in \left\{1,2\right\}, \end{array}$$

(25d)$$\begin{array}{cc} \; & R_{C,i,r,1}+R_{C,i,r,2}\leq C_{F},\;\;\forall i\in \left\{1,2\right\},r\in M, \end{array}$$

(25e)$$\begin{array}{cc} \; & \sum_{r\in M}R_{C,i,r,1}\leq C_{B},\;\;\forall i\in \left\{1,2\right\}, \end{array}$$

(25f)$$\begin{array}{cc} \; & R_{C,i,r,j}\geq 0,\;\;\omega_{i,r,j}>0,\;\;\forall i\in \left\{1,2\right\},\;r\in M,\;j\in \left\{1,2\right\}, \end{array}$$
**4.** Stop if a convergence criterion is satisfied. Otherwise, go back to Step 3 with $\omega^{\prime }\leftarrow \omega$.

## 5. Numerical Results

In this section, we demonstrate numerical results that validate the efficiency of the proposed inter-cluster cooperation scheme. In the simulation, we assume that the channel coefficients $h_{i,r,k}$ and $g_{i,r,k}$ follow independent and identically distributed (i.i.d.) Rayleigh fading distribution, i.e., $h_{i,r,k}∼\mathrm{CN}\left(0,1\right)$ and $g_{i,r,k}∼\mathrm{CN}\left(0,1\right)$. We compare the performance of the proposed cooperation scheme (Section 4) with the following benchmark schemes.

- Perfect backhaul: Two BBUs can perfectly cooperate without any constraint, and each BBU *i* decodes in-cluster messages $W_{i,k}$, k∈K, while treating the other-cluster signals as noise.
- No backhaul (Section 3.1): There are no backhaul links, and hence the BBUs do not exchange any information.
- Conventional cooperation (Section 3.2) with fixed $\tilde{M}$.
- Conventional cooperation (Section 3.2) with optimal $\tilde{M}$.

The sum-rate that is achieved with the perfect backhaul links is given as $R_{\mathrm{sum}}=\sum_{i\in \{1,2\}}R_{\mathrm{sum},i}$, where the sum-rate $R_{\mathrm{sum},i}$ of cluster *i* is given as

(26) $$\begin{array}{c} R_{\mathrm{sum},i}=\log_{2}\det\left(I+P{\left(PA_{\bar{i}}A_{\bar{i}}^{H}+\sigma_{z}^{2}I+\bar{\Omega }\right)}^{-1}A_{i}A_{i}^{H}\right). \end{array}$$

Here, we define the matrices $A_{1}=\left[H_{1};G_{2}\right]$, $A_{2}=\left[G_{1};H_{2}\right]$, and $\bar{\Omega }≜\mathrm{diag}\left({\left\{\omega_{1,r}\right\}}_{r\in M},{\left\{\omega_{2,r}\right\}}_{r\in M}\right)\in C^{2M\times 2M}$ with $\omega_{i,r}$ given as Equation (6). To find the optimal $\tilde{M}$ of the last scheme, we perform an exhaustive search over $\tilde{M}\in \left\{0,1,\ldots ,M\right\}$.

**Figure 2:**
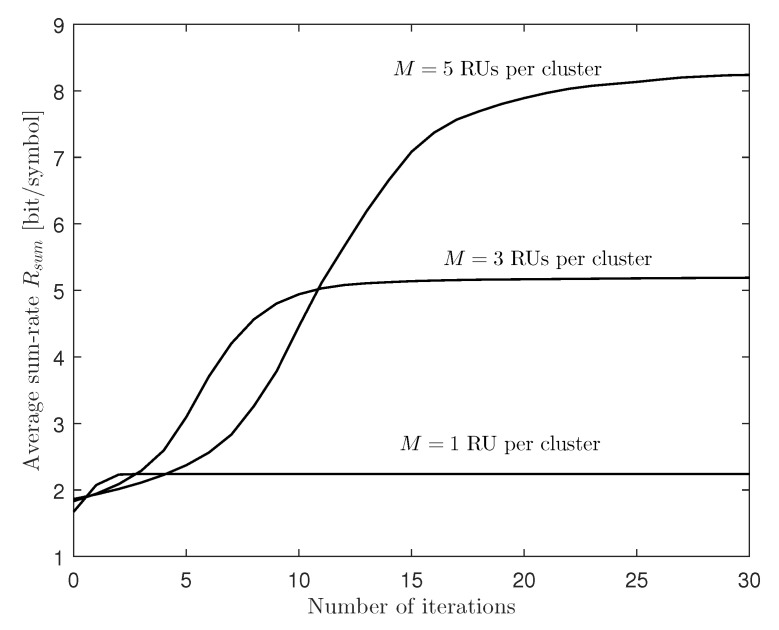
Average sum-rate $R_{\mathrm{sum}}$ versus the number of iterations for a two-cluster C-RAN uplink system with M∈{1,3,5}, K=6, $C_{B}=1$, $C_{F}=2$, and 20 dB SNR.

To investigate the convergence property of the proposed algorithm, Figure 2 plots the average sum-rate $R_{\mathrm{sum}}$ with respect to the number of iterations for a two-cluster C-RAN uplink system with M∈{1,3,5}, K=6, $C_{B}=1$, $C_{F}=2$ and 20 dB SNR. It is observed that, as the network size increases (i.e., the number *M* of RUs increases), more iterations are needed for convergence. However, for all simulated cases, the algorithm converges within a few tens of iterations. In the simulation of the remaining results, we limit the maximum number of iterations to $N_{\max}=30$, which means that Algorithm 1 stops if the updated sum-rate is sufficiently close to the previous sum-rate, or the number of iterations reaches $N_{\max}$.

**Figure 3:**
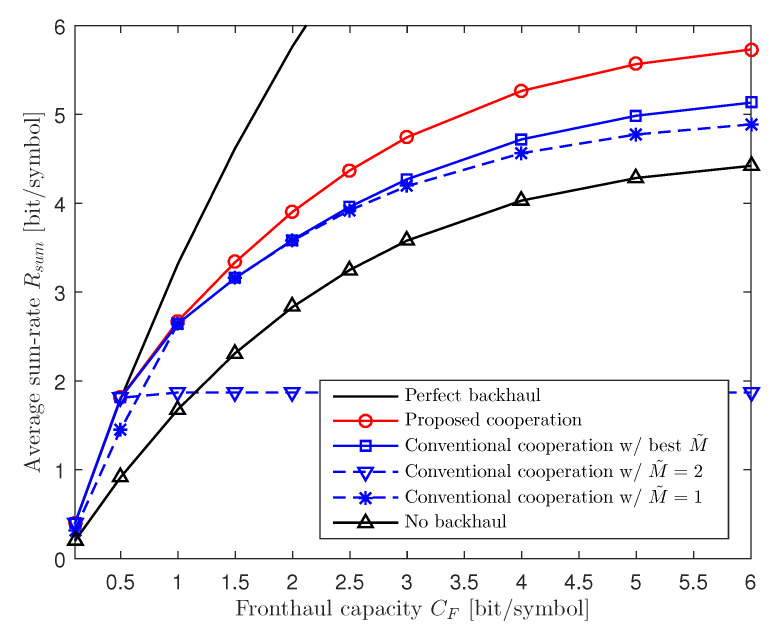
Average sum-rate $R_{\mathrm{sum}}$ versus the fronthaul capacity $C_{F}$ for a two-cluster C-RAN uplink system with M=2, K=6, $C_{B}=1$, and $P/\sigma_{z}^{2}=20$ dB.

In Figure 3, we plot the average sum-rate $R_{\mathrm{sum}}$ versus the fronthaul capacity $C_{F}$ for a two-cluster C-RAN uplink system with M=2, K=6, $C_{B}=1$, and $P/\sigma_{z}^{2}=20$ dB. From the figure, we can see that the performance of the conventional cooperation scheme with fixed $\tilde{M}=M$ can be worse than that of the no cooperation scheme, particularly when the RUs-to-BBU fronthaul links have much larger capacity than the inter-BBU backhaul links (i.e., $C_{F}>C_{B}$). This is because forwarding the quantized signals of many in-cluster RUs to the other BBU on low-capacity backhaul links limits the resolution of the quantized signals and makes the capacity of fronthaul links not fully utilized. In addition, we can achieve a notable gain by adopting the conventional inter-BBU cooperation scheme with the optimal $\tilde{M}$ compared to the no cooperation scheme, and the gain increases with the fronthaul capacity $C_{F}$. However, we should perform an MM algorithm for each possible value $\tilde{M}\in \left\{0,1,\ldots ,M\right\}$ to find the optimal $\tilde{M}$. We note that the proposed scheme can achieve a further gain particularly at large $C_{F}$ without resorting to a discrete search. 

**Figure 4:**
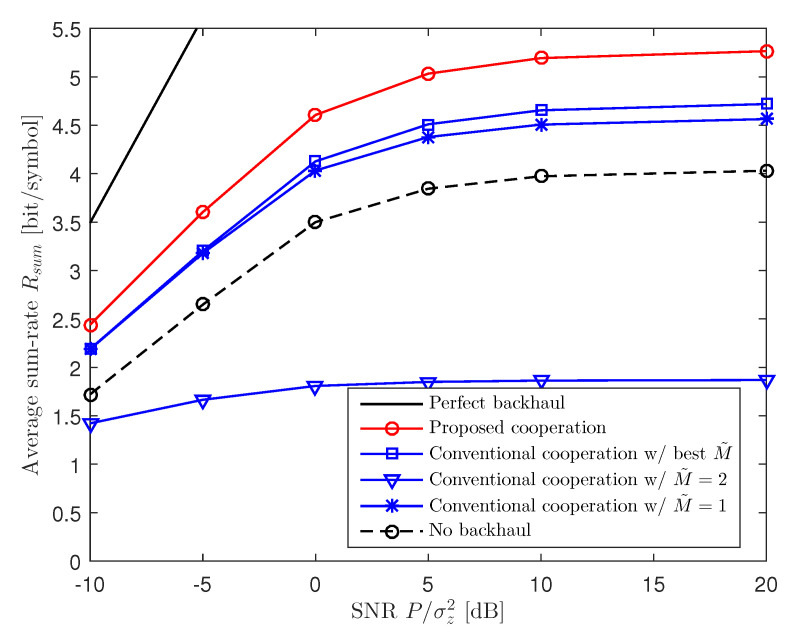
Average sum-rate $R_{\mathrm{sum}}$ versus the SNR $P/\sigma_{z}^{2}$ for a two-cluster C-RAN uplink system with M=2, K=6, $C_{B}=1$, and $C_{F}=4$.

Figure 4 plots the average sum-rate $R_{\mathrm{sum}}$ with respect to the SNR $P/\sigma_{z}^{2}$ for a two-cluster C-RAN uplink system with M=2, K=6, $C_{B}=1$, and $C_{F}=4$. The figure shows that the performance gaps among the schemes increase with the SNR of the uplink channel. This suggests that the importance of inter-BBU cooperation on the backhaul links becomes more significant at high SNRs, since the overall performance will be more interference-limited in that regime. 

**Figure 5:**
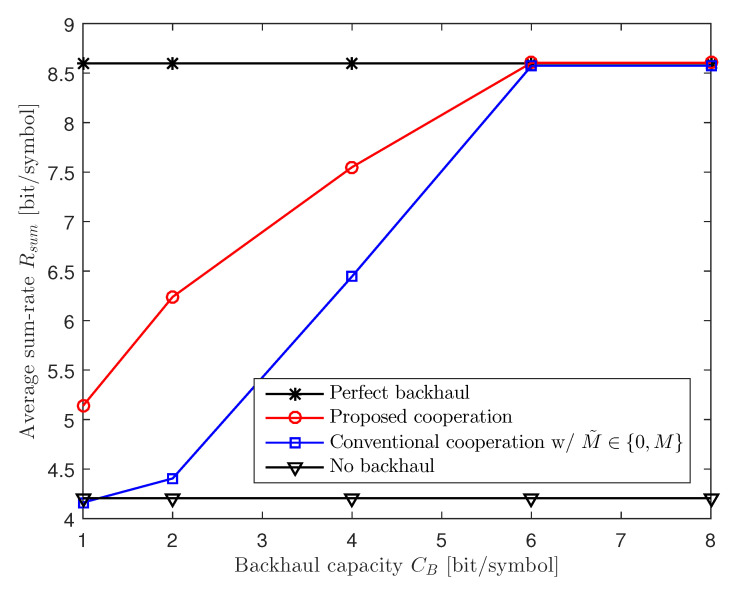
Average sum-rate $R_{\mathrm{sum}}$ versus the backhaul capacity $C_{B}$ for a two-cluster C-RAN uplink system with M=3, K=10, $C_{F}=2$, and 20 dB SNR.

In Figure 5, we investigate the impact of capacity $C_{B}$ of the backhaul links by plotting the average sum-rate with respect to $C_{B}$ for a two-cluster C-RAN uplink system with M=3, K=10, $C_{F}=2$, and 20 dB SNR. For the conventional cooperation scheme, we choose the cooperation level $\tilde{M}$ from $\tilde{M}\in \left\{0,M\right\}$. We can see in the figure that the proposed scheme outperforms the conventional cooperation scheme when the backhaul links do not have enough capacity. However, as the backhaul capacity $C_{B}$ becomes sufficiently large, both the proposed and conventional inter-BBU cooperation schemes achieve the performance of the perfect backhaul scheme.

**Figure 6:**
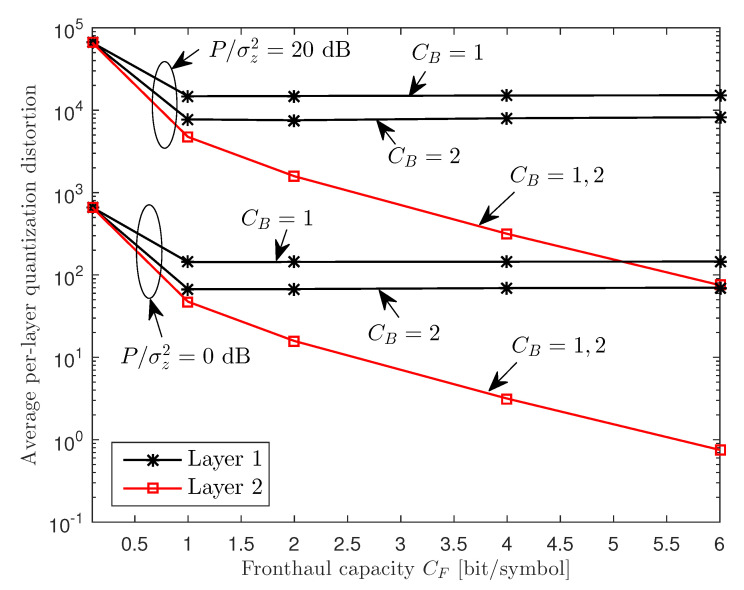
Average per-layer quantization distortions of the proposed scheme (Section 4) versus the fronthaul capacity $C_{F}$ for a two-cluster C-RAN uplink system with M=2, K=6, $C_{B}\in \left\{1,2\right\}$, and $P/\sigma_{z}^{2}=20$ dB.

In Figure 6, we plot the average per-layer quantization distortion of the proposed scheme in Section 4 with respect to the fronthaul capacity $C_{F}$ for a two-cluster C-RAN uplink system with M=2, K=6, $C_{B}\in \left\{1,2\right\}$, and $P/\sigma_{z}^{2}=20$ dB. We define the quantization distortion $D_{j}$ of layer *j*, j∈{1,2}, as $D_{j}≜\sum_{i\in \{1,2\},r\in M}\omega_{i,r,j}$. The figure shows that, as the fronthaul capacity $C_{F}$ increases, the quantization distortion of Layer 2 signals, which are described by both basement and enhancement layers, keeps decreasing. However, the distortion of Layer 1 signals, which are described by only the basement layer, is saturated to a certain level if $C_{F}$ exceeds a threshold value. This is because the basement layer descriptions are transferred on the backhaul links of fixed capacity $C_{B}$.

**Figure 7:**
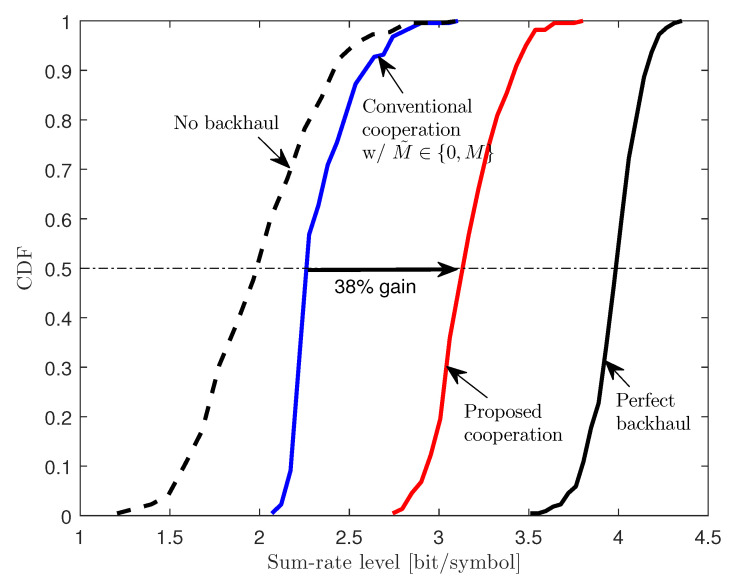
CDFs of sum-rates $R_{\mathrm{sum}}$ of various schemes for a two-cluster C-RAN uplink system with M=2, K=6, $C_{F}=C_{B}=1.25$, and 20 dB SNR.

In Figure 7, we observe the sum-rate cumulative distribution functions (CDFs) of the schemes considered in Figure 5 for a two-cluster C-RAN uplink system with M=2, K=6, $C_{F}=C_{B}=1.25$, and 20 dB SNR. In the figure, we choose $C_{F}=C_{B}=1.25$ bit/symbol to reflect the system parameters of 5G New Radio (NR) [23] and Common Public Radio Interface (CPRI) specification [24]: Bandwidth per component carrier considered in 5G NR is scalable up to 800 MHz [23], and the fronthaul capacity supported by the CPRI specification ranges from 500 Mbit/s to 12 Gbit/s [24]. We focus on a relatively challenging case where the bandwidth and fronthaul capacity are equal to 400 MHz and 500 Mbit/s, respectively, so that the fronthaul capacity $C_{F}$ in bit/symbol is approximated to 1.25. The backhaul capacity is assumed equal to the fronthaul capacity, i.e., $C_{B}=C_{F}$. Figure 7 shows that the proposed cooperation scheme significantly outperforms the conventional cooperation scheme. In particular, in terms of 50%-ile sum-rate, the gain amounts to 38%.

## 6. Conclusions

We studied inter-cluster cooperative reception for the uplink of a two-cluster C-RAN system, where the BBUs of two neighboring clusters communicate with each other via finite-capacity backhaul links with the goal of mitigating the impact of inter-cluster interference signals. To overcome the limitation of conventional cooperation scheme, in which each RU produces a single description, we proposed an improved cooperation strategy, whereby each RU performs layered compression to produce two descriptions, among which only a single description is forwarded to the neighboring BBU and the compression rate allocation is subject to optimization. We tackled the optimization of the proposed cooperation scheme with the goal of maximizing the sum-rate of all the users. Via numerical results, the advantages of the proposed cooperation scheme compared to the baseline schemes with no or conventional inter-cluster cooperation were validated. As future work, we mention the analysis of the proposed cooperation scheme while taking into account the system complexity of successive refinement quantization strategy and the robust design of inter-cluster cooperation strategies in the presence of random packet losses on the fronthaul and backhaul links.
